# Adverse childhood events and the timing of Alzheimer's Disease in Down syndrome

**DOI:** 10.1002/alz.089584

**Published:** 2025-01-09

**Authors:** Emily J Hickey

**Affiliations:** ^1^ University of Wisconsin‐Madison, Madison, WI USA

## Abstract

**Background:**

People with Down syndrome (DS) are genetically at‐risk for Alzheimer’s disease (AD). The age of symptomatic AD in DS varies (late‐40s‐70s). Lifestyle factors are theorized to explain some of this variability. Experiencing adverse childhood events (ACEs) is associated with later‐life cognitive decline and risk for AD in general population samples. Mechanisms underlying this association may involve biological pathways such as systemic inflammation and/or increased mental health conditions such as anxiety and depression, which are themselves risk factors for AD. Little is known about the effect of ACEs on AD pathology (PET Aβ and tau) or symptomology directly, or indirectly through inflammation or mental health in DS.

**Methods:**

We investigated ACEs prevalence in 112 adults with DS (mean age=39.0, range 25‐61; mild ID: 60.7%) and associations with plasma markers of inflammation, informant‐reported mental health, and PET Aβ and tau, controlling for age. Inflammation was measured by a composite of plasma IL‐6 (interleukins), serum IL‐6, serum CRP (C‐reactive protein), and serum sICAM (soluble intercellular adhesion molecule) levels. Mental health problems were measured via the Anxiety, Depression, and Mood Scale (ADAMS), Glasgow Depression Scale‐Caregiver Supplement (CGDS), and Reiss Screen for Maladaptive Behaviors (RSMB). Dementia symptoms were assessed via the Down Syndrome Mental Status Exam (DSMSE).

**Results:**

Eighty percent (n=90) of participants reported at least one ACE and 24% (n=27) reported ≥4. ACEs were positively associated with anxiety (t=2.35, p=.021), depression (t=2.64, p=.010), maladaptive behaviors (t=3.35, p=.001), and plasma inflammatory markers (t=2.40, p=.021), controlling for age. ACEs were not significantly associated with the DSMSE. ACEs were associated with tau (t=‐2.58, p=.013) and at a trend level, with Aβ (t=‐1.95, p=.057), controlling for age, although not in the expected directions.

**Conclusions:**

People with DS are vulnerable to ACEs and experience expected associations between higher ACEs and more mental health problems and systemic inflammation, but ACEs were not related to dementia symptoms. Unexpectedly, ACEs were related to lower PET Aβ and tau relative to age. It is possible that ACEs may be linked to other AD protective factors, such as time spent in the community. Future research should look into pathways that explain this finding.

